# HDAC9 and miR-512 Regulate CAGE-Promoted Anti-Cancer Drug Resistance and Cellular Proliferation

**DOI:** 10.3390/cimb46060311

**Published:** 2024-05-24

**Authors:** Minjeong Yeon, Nayeon Kwon, Jaewhoon Jeoung, Dooil Jeoung

**Affiliations:** Department of Biochemistry, College of Natural Sciences, Kangwon National University, Chuncheon 24341, Republic of Korea; myeon@wistar.org (M.Y.); naabelle@naver.com (N.K.); heyjhw@kangwon.ac.kr (J.J.)

**Keywords:** anti-cancer drug resistance, CAGEs, HDAC9, miR-512

## Abstract

Histone deacetylase 9 (HDAC9) is known to be upregulated in various cancers. Cancer-associated antigens (*CAGEs*) are cancer/testis antigens that play an important role in anti-cancer drug resistance. This study aimed to investigate the relationship between CAGEs and HDAC9 in relation to anti-cancer drug resistance. AGS^R^ cells with an anti-cancer drug-resistant phenotype showed higher levels of CAGEs and HDAC9 than normal AGS cells. CAGEs regulated the expression of HDAC9 in AGS and AGS^R^ cells. CAGEs directly regulated the expression of HDAC9. Rapamycin, an inducer of autophagy, increased HDAC9 expression in AGS, whereas chloroquine decreased HDAC9 expression in AGS^R^ cells. The downregulation of *HDAC9* decreased the autophagic flux, invasion, migration, and tumor spheroid formation potential in AGS^R^ cells. The TargetScan analysis predicted that miR-512 was a negative regulator of HDAC9. An miR-512 mimic decreased expression levels of CAGEs and HDAC9. The miR-512 mimic also decreased the autophagic flux, invasion, migration, and tumor spheroid forming potential of AGS^R^ cells. The culture medium of AGS^R^ increased the expression of HDAC9 and autophagic flux in AGS. A human recombinant CAGE protein increased HDAC9 expression in AGS cells. AGS^R^ cells displayed higher tumorigenic potential than AGS cells. Altogether, our results show that CAGE–HDAC9–miR-512 can regulate anti-cancer drug resistance, cellular proliferation, and autophagic flux. Our results can contribute to the understanding of the molecular roles of HDAC9 in anti-cancer drug resistance.

## 1. Introduction

*Cancer-associated antigens* (*CAGEs*) are first identified in the sera of patients with gastric cancer [1]. It was then detected in the sera of patients with various other cancers [2,3]. CAGEs are also expressed in various diffuse large B cell lymphoma cell lines [4]. The methylation status of its promoter sequences determines *CAGE* expression [5]. CAGEs show oncogenic potential [6].

CAGEs can bind to histone deacetylase 2 (HDAC2), decrease p53 expression, and lead to the resistance of melanoma cells to anti-cancer drugs [7]. It is known that miR-200b can negatively regulate the expression of *CAGEs* and enhance the sensitivity of melanoma cells to microtubule-targeting drugs [8]. CAGEs can bind to glycogen synthase kinase 3β (GSK3β) and increase cyclin D1 expression in melanoma cells [9]. CAGE-derived penta peptide can enhance anti-cancer drug sensitivity by inhibiting the binding of CAGEs to GSK3β [9]. In breast cancer cells, CAGEs can increase autophagic flux and promote resistance to anti-cancer drugs [10]. CAGEs are present in the exosomes of gastric cancer cells and confer resistance to anti-cancer drugs [11]. CAGEs are also present in the exosomes of nasopharyngeal cancer cells and confer resistance to taxol [12].

*Histone deacetylase 9* (*HDAC9*) has been shown to be present in the nucleus, cytoplasm, and deacetylate histone and non-histone substrates [13]. HDAC9 can directly regulate the expression of p53 to promote osteosarcoma cell proliferation [14]. The overexpression of HDAC9 in B cells has been shown to lead to the development of lymphoma in a mouse model [15]. HDAC9 has been found to be upregulated in gastric cancer tissues and retinoblastoma tissues [16,17].

The downregulation of *HDAC9* can inhibit neuronal apoptosis [18]. A selective inhibitor of HDAC9 has been shown to have an apoptotic effect in breast cancer cells [19]. HDAC9 can promote endothelial–mesenchymal transition and contribute to vascular pathology [20]. HDAC9 has been shown to decrease the expression of E-cadherin, an inhibitor of epithelial–mesenchymal transition (EMT) [21].

HDAC9 can inhibit intracellular autophagy by binding to promoter sequences of autophagy-related gene 7 (Atg7), Beclin1, and LC3 [22]. Autophagy is closely related to anti-cancer drug resistance [3,11]. HDAC9 can confer resistance to taxol in triple-negative breast cancer cells [23]. The inhibition of HDAC9 can result in the resistance of AMP-dependent kinase (AMPK)-deficient cells to irradiation [24]. 

We found that CAGEs regulated the expression of HDAC9. We also found that CAGEs could bind to promoter sequences of *HDAC9*. In addition, anti-cancer drug resistance was found to be closely associated with increased autophagic flux. We also showed that HDAC9 was necessary for anti-cancer drug resistance and autophagic flux. *HDAC9* was shown to function as a target of miR-512. miR-512 was found to be able to decrease invasion, migration, and autophagic flux in AGS^R^ cells. miR-512 was also found to enhance the sensitivity of AGS^R^ cells to anti-cancer drugs. Altogether, our results showed a novel mechanism of CAGE-promoted anti-cancer drug resistance. The CAGE–HDAC9–miR-512 loop can be employed for developing anti-cancer drugs.

## 2. Materials and Methods

### 2.1. Materials

We purchased Lipofectamine and PLUS^TM^ reagent from Invitrogen (San Diego, CA, USA). Oligonucleotides, miRNA mimic, and small interfering RNAs (siRNAs) were purchased from the Bioneer Company (Daejeon, Republic of Korea).

### 2.2. Cell Lines and Cell Culture

We purchased human cancer cell lines from the Korea Cell Line Bank (Seoul, Republic of Korea). Cancer cell lines were cultured in Dulbecco’s modified minimal essential medium (DMEM) containing heat-inactivated 10% fetal bovine serum. Anti-cancer drug-resistant AGS^R^ cells were established by the stepwise addition of celastrol to AGS gastric cancer cells. AGS^R△CAGE#5^ and AGS^R△CAGE#7^ cell lines were generated using the CRISPR-Cas9 system. AGS^R^ cells were stably transfected with Cas9, CAGE-targeted sgRNA, and reporter plasmid and selected by hygromycin B.

### 2.3. Colony Formation

The cells were mixed with 0.4% trypan blue staining solution in a 1:1 ratio and counted using a hemocytometer. Two hundred cells were seeded onto 6-well plates and maintained at 37 °C in 5% CO_2_ for 7 days. Colonies were stained with 0.01% crystal violet and counted. 

### 2.4. Cell Viability Determination 

The cancer cells (2 × 10^4^) were plated in 48-well plates. Then, 24 h after incubation, the cells were treated with the anti-cancer drug for 48 h. The cells were treated with 0.5 mg/mL 3-(4,5-dimethylthiazol-2-yl)-2,5-diphenyltetrazolium bromide (MTT) and incubated for 1 h. After removing the solution, MTT formazans were dissolved with DMSO, and the plates were read at 570 nm. GraphPad Prism 7 software was used for the determination of the IC_50_ value.

### 2.5. Invasion Assays

Trypsinized cells (5 × 10^3^) in the serum-free DMEM medium were added to each upper chamber of the transwell with 8-μm pore polycarbonate filter inserts (CoSTAR, Acton, MA, USA). DMEM containing 10% fetal bovine serum was placed in the lower chamber. Incubation was continued at 37 °C for 16 h. The invaded cells were stained and counted as described [11]. 

### 2.6. ChIP Assays

Assays were carried out according to the protocol provided by the manufacturer (Upstate Company). For the detection of the binding of CAGEs to *HDAC9* promoter sequences, specific primers of *HDAC9* promoter-1 sequences [5′-ATTCTGGGGTGTGCTTGTTTTC-3′ (sense) and 5′-ATACTGGCGATTCGCTTCCAA-3′ (antisense)], HDAC9 promoter-2 sequences [5′-CTGGACAGCTGGGTTTGCTG-3′ (sense) and 5′-GAGTTCTTCAGGCTGCTAGGG-3′ (antisense)], and HDAC9 promoter-3 sequences [5′-GACAAAGAAATAACCCCGAAGCA-3′ (sense) and 5′-CAGGAGCTACCCTCGCTGG-3′ (antisense)] were used. 

### 2.7. Tumor Spheroid Forming Potential

The cells were plated (5 × 10^4^ cells/well) in ultralow attachment plates (Corning Inc., Corning, NY, USA) and fed with 0.2 mL of fresh stem cell medium on days 2, 4, and 6. The number of tumor spheroids was counted after 5–14 days of culture. Those larger than 50 μm were counted as tumor spheroids.

### 2.8. Transfection

The transfections were performed with JetPEI^®^ (Polyplus, cat.201-10G, New York, NY, USA) using the protocol provided by the manufacturer (Polyplus). All the transfections were carried out in the presence of a serum. The cells were transfected with siRNA (each at 10 nM) or miR mimic (each at 10 nM) for 24 h. The sequences for miRNA mimics and siRNAs are listed in Supplementary Tables S1 and S2, respectively.

### 2.9. miRNA Extraction and qRT-PCR

Total miRNA was isolated using the miRNeasy Micro Kit (Qiagen, San Diego, CA, USA). The total miRNA was converted into cDNA using a miScript II RT Kit (Qiagen). SYBR Green Master Mix (Qiagen) was used to determine the expression level of miR-512. The expression level of miR-512 was defined based on the threshold (Ct), and relative expression levels were calculated as 2^−(Ct of miR 512)−(Ct of U6)^ after normalization with reference to the expression of U6 small nuclear RNA.

Total RNA was isolated using TRIzol reagent (Thermo Fisher, Waltham, MA, USA). Total RNA was converted into cDNA using the protocol provided by the manufacturer (iNtRon Biotechnology, Kyunggi, Republic of Korea). Quantitative RT-PCR was performed using the synthesized cDNA and an SYBR Green mixture containing the Rox dye (Excel Taq™ 2X Fast Q-PCR Master Mix) (SMOBIO, Hsinchu, Taiwan) in a StepOne^TM^ Real-Time PCR System (Thermo Fisher). PCR conditions were 40 cycles of denaturation for 30 s at 95 °C, annealing for 30 s at 60 °C, and extension for 30 s at 72 °C. The sequences of primers targeting *CAGEs* and *HDAC9* are listed in Supplementary Table S3.

### 2.10. TargetScan Analysis

The identification of miRNAs that can bind to the UTR of *HDAC9* was carried out using the TargetScan program (http://www.targetscan.org, accessed on 28 April 2021), Diana laboratory (http://dianna.imis.athena-innovation.gr, accessed on 28 April 2021), and miRDB (http://mirdb.org, accessed on 28 April 2021).

### 2.11. Immunofluorescence Staining

The cells were fixed with 4% paraformaldehyde and were permeabilized by Triton X-100. After blocking with goat serum (10%) in 0.1% BSA, incubation with anti-LC3 antibody at 4 °C overnight was performed. Next, incubation with anti-rabbit Alexa Fluor 488 (for LC3) secondary antibody was followed. The cells were stained with DAPI and mounted with a mounting medium. 

### 2.12. Immunoblot and Immunoprecipitation

The cell lysates were isolated using lysis buffer (50 mM Tris-HCl, pH 8.0, NP-40 1% (*v*/*v*), 0.1% (*v*/*v*) protease inhibitor mixture (Roche, Singapore), and 200 μM sodium orthovanadate). The cell lysates (20 μg/well) were loaded onto a 10% SDS-PAGE and were transferred onto a PVDF membrane. The following primary antibodies were used for immunoblot: CAGE (MBS2524843, MyBioSource, San Diego, CA, USA), HDAC9 (sc-398003; Santa Cruz, Santa Cruz, CA, USA), AMPKα (AF3194, R&D Systems, Minneapolis, MN, USA), pAMPKα*^Thr172^* (2535S, Cell Signaling, Danvers, MA, USA), PARP (9542S, Cell Signaling), Beclin1 (sc-48341; Santa Cruz), pBeclin1*^Ser15^* (84966S, Cell Signaling), LC3 (12741S, Cell Signaling), Beclin1 (sc-48341, Santa Cruz), MDR1 (12683s, Cell Signaling), IgG (sc-2025, Santa Cruz), FLAG (F3166, Sigma, New York, NY, USA), and p62 (ab56416, Abcam, Cambridge, UK). For immunoprecipitation, cell lysates were incubated with each antibody (2 μg/mL) with constant agitation at 4 °C. The immunocomplexes were precipitated with protein A/G-Sepharose (Sigma) and analyzed by immunoblot. The following secondary antibodies were used in this study: anti-mouse HRP secondary antibody (31430, Invitrogen, Carlsbad, CA, USA), anti-goat HRP secondary antibody (31402, Invitrogen), anti-rabbit HRP secondary antibody (ADI-SAB-300-J, Enzo, Farmingdale, NY, USA), and anti-rabbit Alexa Fluor 488 secondary antibody (A11008, Invitrogen).

### 2.13. Luciferase Activity Assays

The 3′ UTR of HDAC9 (381 bp) was cloned into the *Xba*I site of the pGL3 luciferase plasmid. The mutant pGL3–3′ UTR–HDAC9 was made using the QuikChange site-directed mutagenesis kit (Stratagene, La Jolla, CA, USA). A luciferase activity assay was carried out according to the standard procedures [11]. PCR-amplified full-length human *HDAC9* promoter and deletion constructs were cloned into the pGL2 basic luciferase plasmid. 

### 2.14. Expression and Purification of CAGE Proteins

Human full-length CAGE proteins were purified as described [11].

### 2.15. In Vivo Tumorigenic Potential

AGS or AGS^R^ cells (5 × 10^6^) were injected subcutaneously into the dorsal flank area of the athymic nude mice to induce the formation of tumors. Animal experiments were carried out according to the guidelines of the Korean Council for the Care and Use of Animals in Research, approved by the Institutional Animal Care and Use Committee (IACUC) of Kangwon National University, and in compliance with ARRIVE guidelines. The animals were kept under standard housing conditions (20~26 °C, 150~300 lux, and 40~60% humidity) with a 14–10-h light–dark period. The animals were allowed free access to food and water. The tumor volume (0.5 × length × width^2^) was calculated. Animal euthanasia was carried out using CO_2_ gas at a 30–70% displacement rate of the cage volume/min using a flow meter according to the American Veterinary Medical Association (AVMA) euthanasia guideline43. 

### 2.16. TCGA Dataset Analysis

UALCAN online software (https://ualcan.path.uab.edu/, accessed on 28 February 2024) was used for TCGA dataset analysis (reference needed). Briefly, TCGA level 3 RNA-seq data were used to analyze gene expression values, and transcripts per million (TPM) was used as the measure of expression in this software. Box plot graphs were generated using Highcharts in this software. The values from primary tumors were classified based on the patients’ clinical data. The tumor grades were categorized from 1 to 4 according to the level of tumor cell differentiation. The cancer stages were categorized from 1 to 4 according to pathologic information based on AJCC. GraphPad Prism was used to modify the graphs from UALCAN software.

### 2.17. Statistical Analysis

GraphPad Prism software (Version 7) was used. The data are presented as means ± standard error of the mean (S.E.M.). The Student’s *t*-test was employed for statistical analysis. Statistical significance was set to *p* < 0.05.

## 3. Results

### 3.1. Anti-Cancer Drug-Resistant Gastric Cancer Cells Show Increased Expression of CAGEs and HDAC9

**Figure 1:**
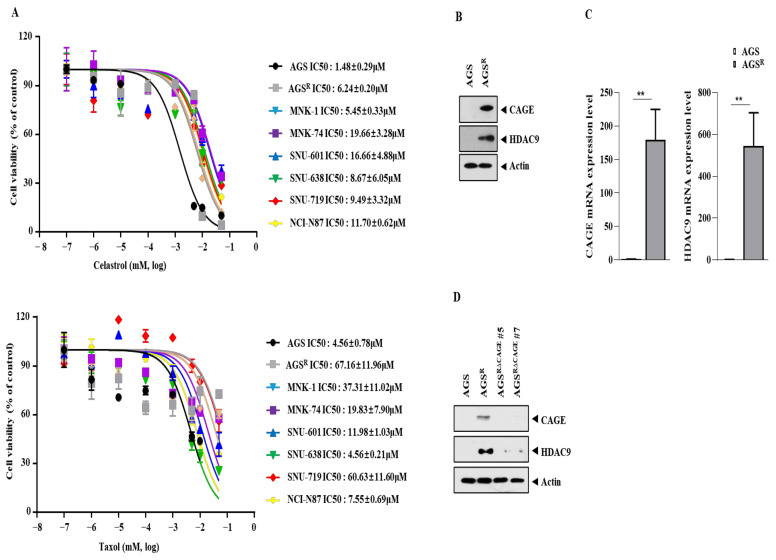
AGS^R^ cells show increased expression of CAGEs and HDAC9. (**A**) Each cancer cell line was treated with various concentrations of celastrol or taxol for 48 h. The sensitivity of each cancer cell line to the indicated anti-cancer drug was determined by an MTT assay. The IC_50_ value was determined by GraphPad Prism 7 Software. Average values of three independent experiments are shown. (**B**) Representative images are shown. The uncropped blots are shown in Supplementary Materials. (**C**) qRT-PCR was performed. **, *p* < 0.01. (**D**) Immunoblot was performed. Representative images were shown.

The sensitivities of various human gastric cancer cell lines to anti-cancer drugs were determined. AGS cells were found to be the most sensitive to celastrol and taxol (Figure 1A). This indicated the relevance of AGS cells as parental anti-cancer drug-sensitive cancer cells. Compared to AGS cells, AGS^R^ cells showed increased resistance to celastrol and taxol (Figure 1A). AGS^R^ cells also showed higher expressions of CAGEs and HDAC9 than AGS cells (Figure 1B). In addition, AGS^R^ cells showed a higher expression of *HDAC9* mRNA than AGS cells (Figure 1C). CRISPR/Cas-9 was used to establish AGS^R^ cells that displayed stable downregulation of *CAGEs* (AGS ^R△CAGE#5^ and AGS^R △CAGE#7^). AGS ^R△CAGE#5^ and AGS^R △CAGE#7^ cell lines showed lower levels of HDAC9 than AGS^R^ cells (Figure 1D). These results imply the existence of a close relationship between CAGEs and HDAC9. 

### 3.2. CAGEs Regulate the Expression of HDAC9

**Figure 2:**
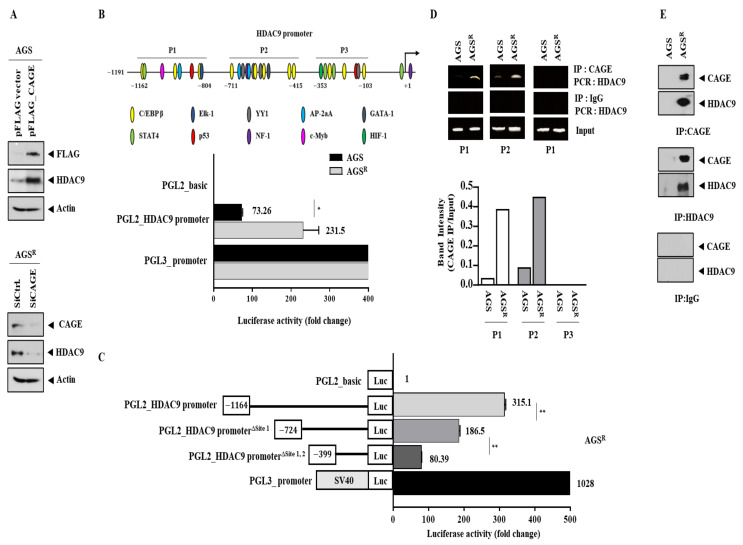
CAGEs directly regulate expression of HDAC9. (**A**) Immunoblot was performed (upper) 48 h after transfection with indicated construct (each at 1 μg). AGS^R^ cells were transfected with indicated siRNA (each at 10 nM). Immunoblot was performed (lower) 48 h after transfection. Representative images are shown. (**B**) Promoter sequences of *HDAC9* contain potential binding sites for various transcription factors. *, *p* < 0.05. (**C**) Luciferase activity assays were performed 48 h after transfection with indicated construct (each at 1 μg). **, *p* < 0.01. (**D**) ChIP assays were performed as described. (**E**) Immunoprecipitation was performed as described. Isotype-matched IgG (2 μg/mL) was also used for immunoprecipitation. Representative images are shown.

Since AGS^R^ cells showed increased expression levels of CAGEs and HDAC9, we examined whether CAGEs could affect the expression of HDAC9. The overexpression of *CAGEs* increased the expression of HDAC9 in AGS cells, while the downregulation of *CAGEs* in AGS^R^ cells decreased the expression of HDAC9 (Figure 2A). We then examined whether increased expression of HDAC9 occurred at the transcriptional level. *HDAC9* promoter sequences contained three potential binding sites for various transcriptional factors (Figure 2B). AGS^R^ cells showed higher luciferase activity associated with *HDAC9* promoter sequences than AGS cells (Figure 2B). This indicates that the increased expression of *HDAC9* occurs at the transcriptional level. The promoter luciferase activity assays showed that the promoter sites 1 and 2 (P1 and P2) of *HDAC9* were both necessary for the increased expression of *HDAC9* in AGS^R^ cells (Figure 2C). The deletion of site 1 decreased luciferase activity associated with the wild-type *HDAC9* promoter (Figure 2C). The deletion of both site 1 and site 2 further decreased luciferase activity associated with the wild-type *HDAC9* promoter (Figure 2C). ChIP assays showed that CAGEs could bind to the promoter sequences of HDAC9 (Figure 2D). CAGE proteins could also bind to HDAC9 proteins in AGS^R^ cells (Figure 2E). Thus, CAGEs can bind to the promoter sequences of *HDAC9* to exert direct regulation of HDAC9 expression. Transfection with the *HDAC9* promoter luciferase construct into AGS^R△CAGE#5^ or AGS^R△CAGE#7^ cells might provide a clue about the possibility of the direct regulation of *HDAC9* expression by *CAGEs*.

### 3.3. HDAC9 Is Necessary for Increased Autophagic Flux in AGS^R^ Cells

**Figure 3:**
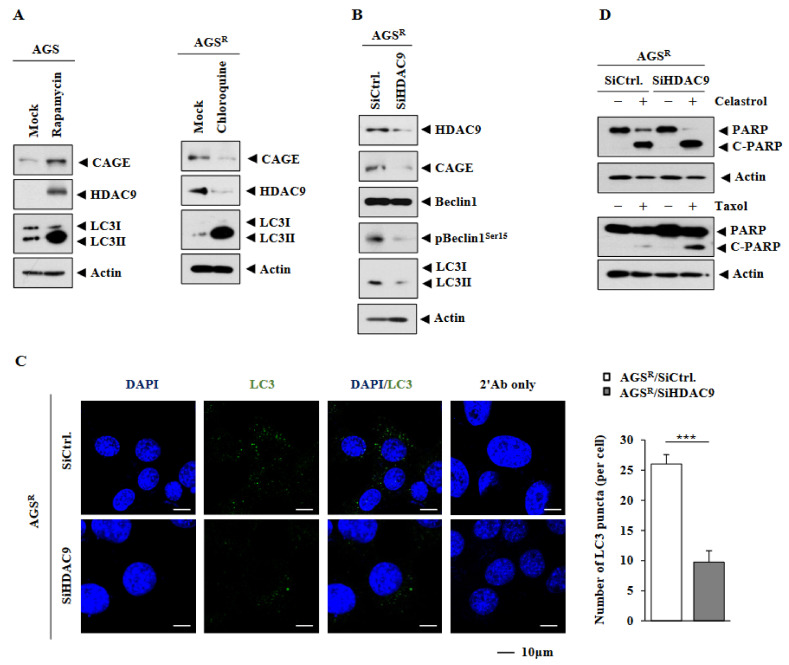
HDAC9 is necessary for increased autophagic flux in AGS^R^ cells. (**A**) AGS cells were treated without or with rapamycin (5 μM) for 24 h (left). AGS^R^ cells were treated without or with chloroquine (100 μM) for 24 h (right). Representative images are shown. (**B**) Immunoblot was performed 48h after transfection with the indicated siRNA (each at 10 nM). Representative images are shown. (**C**) Same as (**B**), except that immunofluorescence staining was performed. ***, *p* < 0.001. Representative images are shown. (**D**) Here, 24 h after transfection with each siRNA (10 nM), AGS^R^ cells were treated with celastrol (1 μM) or taxol (1 μM) for 24 h. Representative images of three independent experiments are shown. The uncropped blots are shown in Supplementary Materials.

CAGEs induced anti-cancer drug resistance by decreasing the expression of p53 in melanoma cells [7]. Anti-cancer drug resistance induced by CAGEs was accompanied by increased autophagic flux [11]. The overexpression of *HDAC9* promotes cancer cell proliferation by suppressing the expression of p53 [14]. Therefore, we examined whether increased autophagic flux could affect the expression of HDAC9. Rapamycin, an inducer of autophagy, increased the expression levels of CAGEs, HDAC9, and LC3II in AGS cells (Figure 3A). Chloroquine, an inhibitor of autophagy, decreased the expression levels of CAGEs and HDAC9 in AGS^R^ cells (Figure 3A). The downregulation of *HDAC9* decreased the expression levels of CAGEs, pBeclin1^Ser15^, and LC3II in AGS^R^ cells (Figure 3B). Increased expression of pBeclin1^Ser15^ and LC3II is known to be associated with anti-cancer drug resistance [11]. Immunofluorescence staining showed that the downregulation of *HDAC9* decreased LC3 puncta in AGS^R^ cells (Figure 3C). However, the downregulation of *HDAC9* increased the expression of cleaved PARP in AGS^R^ cells in response to celastrol and taxol (Figure 3D). Thus, CAGEs and HDAC9 are likely to promote anti-cancer drug resistance by regulating autophagic flux.

### 3.4. Downregulation of HDAC9 Decreases Invasion, Migration, and Tumor Spheroid Forming Potential of AGS^R^ Cells

**Figure 4:**
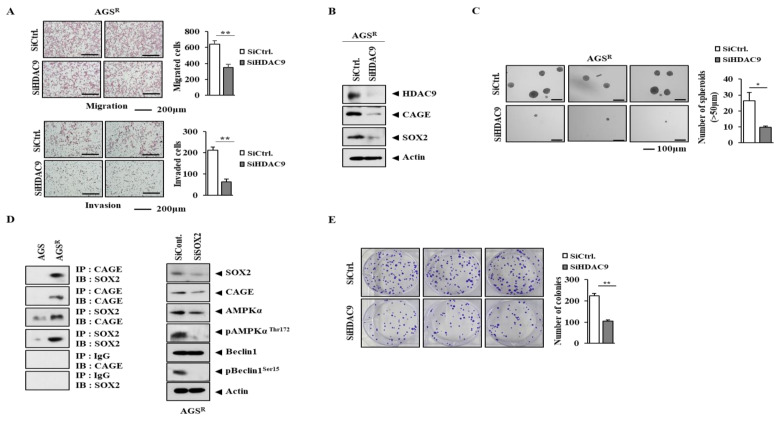
HDAC9 is necessary for invasion/migration and tumor spheroid forming potential of AGS^R^ cells. (**A**) Invasion and migration assays of AGS^R^ cells (10^4^ cells) were carried out 48 h after transfection with each siRNA (10 nM). **, *p* < 0.01. (**B**) Immunoblot was performed 48 h after transfection. Representative images of three independent experiments are shown. The uncropped blots are shown in Supplementary Materials. (**C**) Tumor spheroid formation assays of AGS^R^ cells were carried out 48 h after transfection. *, *p* < 0.05. Those larger than 50 μm were counted as tumor spheroids. (**D**) Cell lysates were subjected to immunoprecipitation (left). Cell lysates were subjected to immunoblot analysis 48 h after transfection. Representative images are shown. Isotype-matched IgG (2 μg/mL) was also used for immunoprecipitation. (**E**) AGS^R^ cells (200 cells) were subjected to colony formation assays 48 h after transfection. **, *p* < 0.01.

The downregulation of *HDAC9* decreased the invasion and migration potential of AGS^R^ cells (Figure 4A). Cancer stem cell-like properties are known to be closely related to increased autophagic flux [11,12]. The downregulation of *HDAC9* also decreased SOX2, a marker of cancer stemness, in AGS^R^ cells (Figure 4B). In addition, the downregulation of *HDAC9* decreased the tumor spheroid forming potential of AGS^R^ cells (Figure 4C). AGS^R^ cells showed binding of CAGEs to SOX2 (Figure 4D). The downregulation of SOX2 decreased autophagic flux in AGS^R^ cells (Figure 4D). The downregulation of *HDAC9* decreased the colony forming potential of AGS^R^ cells (Figure 4E). It would be interesting to examine whether the CAGE–HDAC9 complex could bind to the promoter sequences of SOX2 in the future.

### 3.5. miR-512 Directly Regulates the Expression of HDAC9

**Figure 5:**
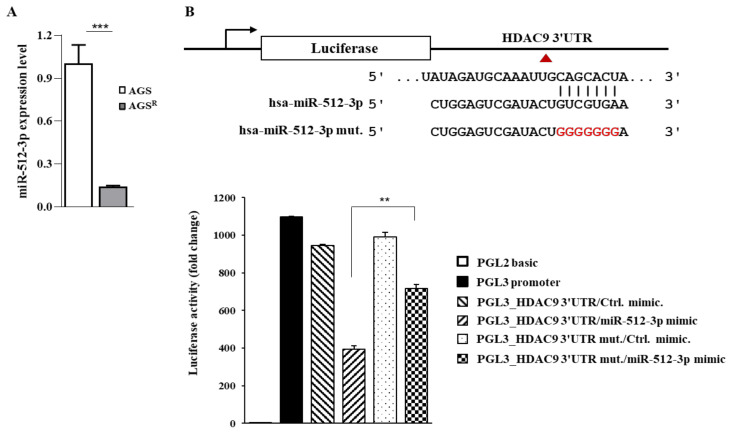
miR-512 directly regulates the expression of HDAC9. (**A**) Cell lysates were subjected to qRT-PCR. ***, *p* < 0.001. (**B**) Wild-type Luc-HDAC9 3′-UTR or mutant Luc-HDAC9 3′-UTR (each at 1 μg) was transfected, along with the indicated mimic (each at 10 nM), into AGS^R^ cells. Luciferase activity assays were performed 48 h after transfection. **, *p* < 0.01. Luciferase activity assays were performed as described. Red color denotes mutant sequences.

Next, we aimed to identify a regulator of HDAC9. miR-512 was predicted to be a negative regulator of *HDAC9* by a TargetScan analysis. AGS^R^ cells showed a lower expression of miR-512 than AGS cells (Figure 5A). miR-512 mimic decreased luciferase activity associated with the wild-type 3’ UTR of *HDAC9*. However, it did not affect luciferase activity associated with the mutant 3’ UTR of *HDAC9* (Figure 5B). Therefore, miR-512 could directly regulate the expression of HDAC9 in AGS^R^ cells. 

### 3.6. miR-512 Decreases Autophagic Flux, Invasion/Migration, and Cellular Proliferation but Enhances Sensitivity to Anti-Cancer Drugs

**Figure 6:**
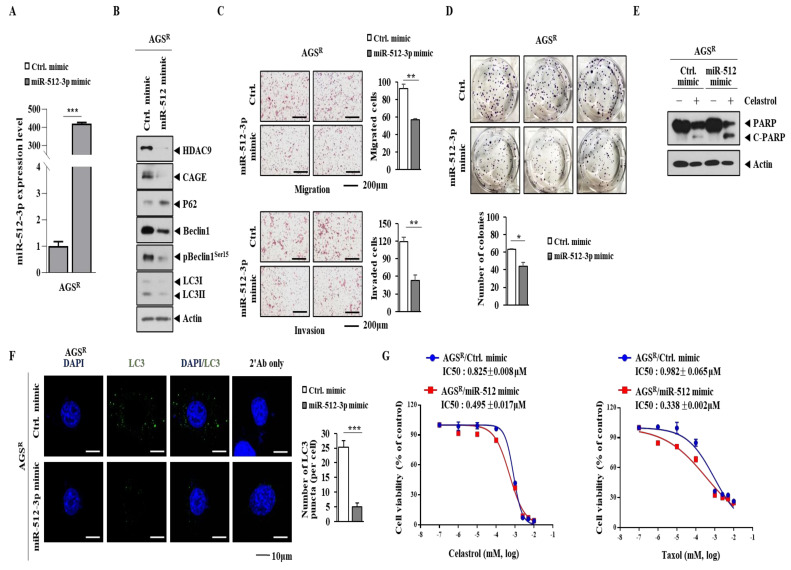
miR-512 decreases autophagic flux, invasion/migration, and cellular proliferation but enhances sensitivity to anti-cancer drugs. (**A**) qRT-PCR was performed 48 h after transfection with indicated mimic (each at 10 nM). ***, *p* < 0.001. (**B**) Same as (**A**), except that immunoblot was performed. Representative images are shown. (**C**) Same as (**A**), except that invasion/migration potential assays were performed. **, *p* < 0.01. (**D**) Colony forming potential assays were performed. Two hundred cells were subjected to colony forming potential assays 48 h after transfection. *, *p* < 0.01. (**E**) AGS^R^ cells were transiently transfected with indicated siRNA (each at 10 nM). Then, 24 h after transfection with each mimic (10 nM), cells were treated with celastrol (1 μM) or taxol (1 μM) for 24 h, followed by immunoblot. Representative images are shown. (**F**) Immunofluorescence staining was performed 48 h after transfection with the indicated mimic (each at 10 nM). ***, *p* < 0.001. (**G**) Here, 24 h after transfection with each mimic (each at 10 nM), cells were treated with various concentrations of celastrol or taxol for 24 h. MTT assays were then performed. Average values of three independent experiments are shown. GraphPad Prism statistics program (GraphPad Prism Software, Version 7) was used to determine IC_50_ value.

The overexpression of miR-512 mimic (Figure 6A) decreased the expression levels of CAGEs, HDAC9, and LC3II but increased the expression of p62 in AGS^R^ cells (Figure 6B). The overexpression of miR-512 mimic also decreased the invasion and migration potential of AGS^R^ cells (Figure 6C). In addition, the overexpression of miR-512 mimic decreased the colony forming potential (Figure 6D) and LC3 puncta in AGS^R^ cells (Figure 6F). However, the overexpression of miR-512 mimic increased the cleavage of PARP in AGS^R^ cells (Figure 6E). In addition, miR-512 mimic enhanced the sensitivity of AGS^R^ cells to both celastrol and taxol (Figure 6G).

### 3.7. Soluble Factors Regulate HDAC9 Expression

**Figure 7:**
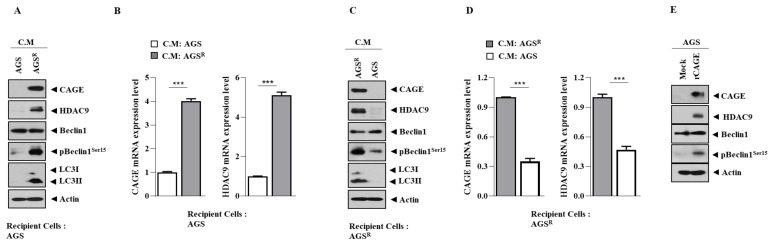
Soluble factors regulate the expression of HDAC9. (**A**) Culture medium of AGS^R^ cells was added to AGS cells. Immunoblot was performed 24 h after addition. Representative images are shown. C.M. denotes culture medium. (**B**) qRT-PCR was performed. ***, *p* < 0.001. (**C**) Culture medium of AGS cells was added to AGS^R^ cells. Representative images are shown. (**D**) Same as (**C**), except that qRT-PCR was performed. ***, *p* < 0.001. (**E**) AGS cells were treated with human recombinant CAGE protein (1 μg/mL) for 24 h. Representative images are shown.

We examined whether soluble factors could regulate the expression of HDAC9 in gastric cancer cells. We used a culture medium for this purpose. When the culture medium of AGS^R^ cells was added to AGS cells, it increased the expression levels of HDAC9 and CAGEs (Figure 7A,B). However, the culture medium of AGS cells decreased the expression levels of HDAC9 and CAGEs in AGS^R^ cells (Figure 7C,D). Human recombinant CAGE proteins increased HDAC9 expression in AGS cells (Figure 7E). These results suggest that the exosomes of AGS^R^ cells could promote anti-cancer drug resistance in AGS cells.

### 3.8. AGS^R^ Cells Display Tumorigenic Potential

**Figure 8:**
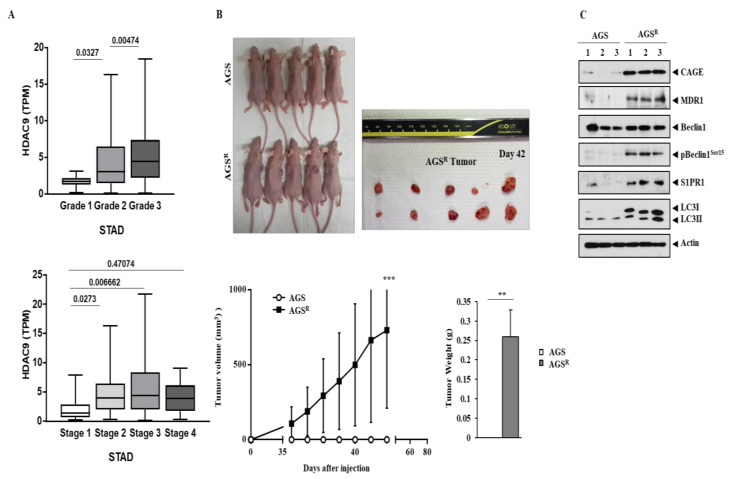
AGS^R^ cells display tumorigenic potential. (**A**) TCGA database shows RNA sequencing analysis of gastric cancer tissues with different grades. High expression level of *HDAC9* is positively associated with poor differentiation and advanced stage of gastric cancer. Grade 1 (*n* = 12): well-differentiated; Grade 2: moderately differentiated; Grade 3: poorly differentiated (*n* = 246); Stage 1 (*n* = 18); Stage 2 (*n* = 123); Stage 3 (*n* = 169); and Stage 4 (*n* = 41). TPM denotes transcripts per million. STAD denotes stomach adenocarcinoma. UALCAN online software (https://ualcan.path.uab.edu/, accessed on 28 February 2024) was used for TCGA database analysis. (**B**) Indicated cancer cells (each at 5 × 10^6^ cells) were injected into dorsal flanks of athymic nude mice. Each experimental group comprised five athymic nude mice. **, *p* < 0.01 and ***, *p* < 0.001. (**C**) Immunoblot was performed using tumor tissue lysates. Numbers denote tissues of tumor (AGS^R^) or corresponding normal tissue (AGS). Representative images of three independent experiments are shown.

The TCGA database analysis showed that a high expression level of *HDAC9* was positively associated with a poorly differentiated state of gastric cancer (Figure 8A). A high level of *HDAC9* was found to be positively associated with an advanced stage of gastric cancer (Figure 8A). AGS^R^ cells, but not AGS cells, displayed tumorigenic potential (Figure 8B). Thus, anti-cancer drug resistance is accompanied by an enhanced tumorigenic potential. Tumor tissues derived from AGS^R^ cells, but not corresponding normal tissues of AGS cells, showed expression of CAGEs, MDR1, pBeclin1^Ser15^, S1PR1, and LC-3II (Figure 8C). It is probable that anti-cancer drug resistance is accompanied by an enhanced autophagic flux.

## 4. Discussion

The overexpression of HDAC9 can enhance the tumorigenic potential of non-small cell lung cancer cells [25]. A high expression of HDAC9 contributes to poor overall survival of patients with hepatocellular carcinomas [26]. HDAC9 expression has been reported to be upregulated in gastric cancer tissues [27]. It would be interesting to examine HDAC9 expression in the sera of gastric cancer patients in the future. Bioinformatics analysis revealed that a high expression of *HDAC9* was correlated with poor survival in patients with gastric cancers [27]. HDAC9 overexpression contributes to the pathogenesis of retinoblastoma. It is related to a poor prognosis [28]. A high expression of HDAC9 is positively related to stemness but negatively related to differentiation markers in hepatocellular carcinomas [29]. These reports suggest that HDAC9, just like CAGEs, can serve as a prognostic marker of cancers.

We found that the expression of HDAC9 in AGS^R^ cells was increased compared to that in AGS cells. It is necessary to further examine the expression levels of other HDACs in AGS^R^ cells. HDAC1 andHDAC2 are known to contribute to the pathogenesis of gastric carcinogenesis by interacting with phosphoribosylaminoimidazole carboxylase and phosphoribosylaminoimidazole succinocarboxamide synthetase (PAICS) [30]. The downregulation of *PAICS* can enhance the sensitivity of gastric cancer cells to cisplatin and inhibit gastric cancer cell growth [30]. A high expression level of *HDAC1/2* is closely associated with a poor prognosis in gastric cancer [31]. The overexpression of *HDA1* and *HDAC2* is closely related to gastric cancer progression [32]. In the present study, we found an increased expression of HDAC6 in AGS^R^ cells compared to AGS cells. HDAC6 inhibition can lead to apoptosis in gastric cancer cells [33]. HDAC6 is known to play an essential role in autophagy [34]. It will be interesting to examine the role of HDAC6 in anti-cancer drug resistance in the future.

CAGEs were found to be able to bind to the promoter sequences of *HDAC9* (Figure 2B). This indicates that CAGEs could play a role as a transcription factor. The promoter sequences of *HDAC9* contain potential binding sites for various transcription factors. It is necessary to further examine the relationship between CAGEs and these transcription factors.

Rapamycin, an inducer of autophagy, was found to increase the expression of HDAC9 in AGS cells (Figure 3A). Chloroquine, an inhibitor of autophagy, showed opposite effects on CAGEs and HDAC9 in AGS^R^ cells (Figure 3A). These results suggest a role of *HDAC9* in autophagy. Since the downregulation of *HDAC9* enhanced the cleavage of PARP in AGS^R^ cells (Figure 3D), the overexpression of *HDAC9* might increase the expression of anti-apoptotic proteins. Many reports have suggested the existence of an inverse relationship between enhanced autophagic flux and apoptosis [10,11]. 

The interaction between CAGEs and HDAC9 has not been previously reported. Identifying the domain of CAGEs that is necessary for binding to HDAC9 may help to understand the mechanisms associated with CAGE-promoted anti-cancer drug resistance. It is also necessary to identify the domain of HDAC9 that is necessary for conferring resistance to anti-cancer drugs. In addition, it is necessary to identify downstream targets of HDAC9 to improve our understanding of CAGE-promoted anti-cancer drug resistance. Identifying the molecular network involving CAGEs and HDAC9 may also be helpful for achieving a better understanding of anti-cancer drug resistance. 

miR-512 serves as a target of circular RNA circRPPH1. It inhibits breast cancer progression [35]. The downregulation of miR-512 can suppress the metastasis of colorectal cancer [36]. The overexpression of miR-512 can enhance sensitivity to cisplatin in ovarian cancer cells [37]. The overexpression of miR-512 can decrease the expression of Mcl-1, resulting in the apoptosis of gastric cancer cells [38]. miR-512 can suppress the progression of Epstein–Barr Virus-associated gastric cancer [39]. miR-512 targets JAG1, which is necessary for the negative regulatory effect of exosomes on metastasis of glioblastoma [40]. These reports suggest that miR-512 can regulate anti-cancer drug resistance in association with autophagy. Since miR-512 directly regulates the expression of *HDAC9* (Figure 5), it is necessary to examine whether CAGEs and HDAC9 can directly regulate the expression of miR-512. It is also necessary to examine the possibility of the binding of CAGEs to the promoter sequence of miR-512.

The TargetScan predicted that miR-512 was a negative regulator of *HDAC9*. AGS^R^ cells showed a lower expression of miR-512 than AGS cells (Figure 5A). *HDAC9* was shown to be directly regulated by miR-512 (Figure 5B). miR-512 can enhance the sensitivity of retinoblastoma cells to cisplatin by promoting apoptosis [41]. miR-512 mimic enhanced the sensitivity of AGS^R^ cells to both celastrol and taxol (Figure 6F). miR-512 mimic is likely to increase caspase-3 activity while decreasing the expression of anti-apoptotic proteins in response to anti-cancer drugs. It is reasonable that miR-512 mimic can increase the number of apoptotic cancer cells in response to anti-cancer drugs. Many other miRNAs are predicted to bind to the 3′ UTR of *HDAC9* based on the TargetScan analysis (Supplementary Figure S1). It will be necessary to examine the roles of these miRNAs in anti-cancer drug resistance. 

We have previously reported that CAGEs can form a negative feedback loop with miR-181b and promote anti-cancer drug resistance in gastric cancer cells [11]. CAGEs can directly regulate the expression of sphingosine-1-phosphate receptor 1 (S1PR1) by binding to the promoter sequences of S1PR1 [11]. miR-181b was shown to act as a direct regulator of S1PR1. Thus, the CAGE–S1PR1–miR–181b loop may provide clues to understanding the mechanism of CAGE-promoted anti-cancer drug resistance. It is necessary to examine the role of S1PR1 for a better understanding of CAGE-promoted anti-cancer drug resistance. The TargetScan analysis predicted miRNAs that can bind to the 3′ UTR of *CAGEs* (Supplementary Figure S1). These miRNAs may regulate the expression of CAGEs and anti-cancer drug resistance.

SOX2 is closely related to cancer stemness-related features [42,43]. Autophagy activation contributes to cancer stem cell-like properties [44]. We found that AGS^R^ cells displayed higher autophagic flux than AGS cells. Cancer stem cell-like properties are known to contribute to chemotherapy resistance in breast cancer cells [45]. It would be interesting to examine whether CAGEs can bind to the promoter sequences of SOX2 in the future. A high expression of SOX2 can promote the resistance of melanoma cells to anti-programmed death ligand-1 (PD-L1) therapy [46]. 

We found that the culture medium of AGS^R^ cells increased HDAC9 expression and autophagic flux in AGS cells (Figure 7A). The exosomes of AGS^R^ cells are likely to increase HDAC9 expression in AGS cells. Thus, the exosomes of AGS^R^ cells may confer resistance to anti-cancer drugs by promoting autophagic flux. Future studies should examine the presence of CAGEs and HDAC9 in the exosomes of AGS^R^ cells.

Cisplatin resistance is accompanied by an increased level of PD-L1 in non-small cell lung cancer cells [47]. AGS^R^ cells might express a higher level of PD-L1 than AGS cells. It will be interesting to examine the resistance of AGS^R^ cells to cisplatin. 

## 5. Conclusions

We provided a novel mechanism of CAGE-promoted anti-cancer drug resistance. Further studies on HDAC9 and miR-512 are necessary for a better understanding of CAGE-promoted anti-cancer drug resistance in relation to autophagy. It would be also necessary to examine whether CAGEs and HDAC9 can promote anti-cancer drug resistance in other gastric cancer cell lines by establishing various anti-cancer drug-resistant cell lines. Although AGS^R^ cells showed resistance to chemotherapeutic drugs, whether AGS^R^ cells are resistant to immunotherapeutic reagents such as anti-PD-L1 antibody remains unclear. This merits further study. It is necessary to determine the oncogenic and metastatic potential of AGS^R^ cells. It is also necessary to examine whether AGS^R^ cells can display in vivo anti-cancer drug resistance. The roles of *CAGEs*, *HDAC9*, and miR-512 in in vivo anti-cancer drug resistance of AGS^R^ cells also merit further study.

## Data Availability

All data generated or analyzed during this study are included in this published article and its Supplementary Information Files. The original contributions presented in this study are included in the article/Supplementary Materials. Further inquiries can be directed to the corresponding author.

## Supplementary Materials

The following supporting information can be downloaded at: https://www.mdpi.com/article/10.3390/cimb46060311/s1.
