# Supplementary material for: HDAC9 and miR-512 Regulate CAGE-Promoted Anti-Cancer Drug Resistance and Cellular Proliferation
Source: Curr Issues Mol Biol. 2024 May 24;46(6):5178–93. doi: 10.3390/cimb46060311 (PMC11201674; doi:10.3390/cimb46060311)
Supplement: Supplementary file 1 [file cimb-46-00311-s001.zip › cimb-2983796-supplementary.pdf]

Figure S1

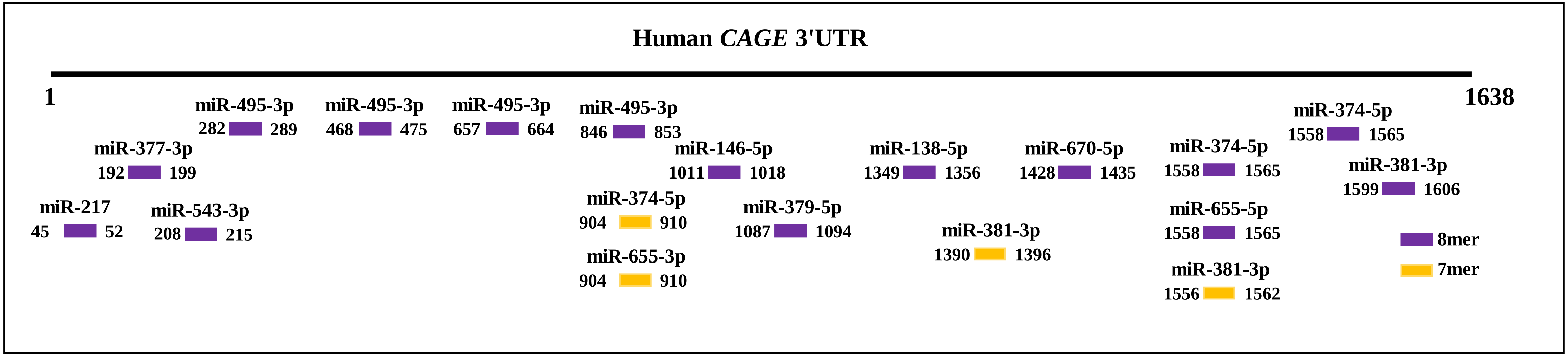
Human CAGE 3'UTR
1
1638
miR-495-3p
282 289
miR-495-3p
468 475
miR-495-3p
657 664
miR-495-3p
846 853
miR-374-5p
1558 1565
miR-377-3p
192 199
miR-146-5p
1011 1018
miR-138-5p
1349 1356
miR-670-5p
1428 1435
miR-374-5p
1558 1565
miR-381-3p
1599 1606
miR-217
45 52
miR-543-3p
208 215
miR-374-5p
904 910
miR-379-5p
1087 1094
miR-381-3p
1390 1396
miR-655-5p
1558 1565
8mer
7mer
miR-655-3p
904 910
miR-381-3p
1556 1562

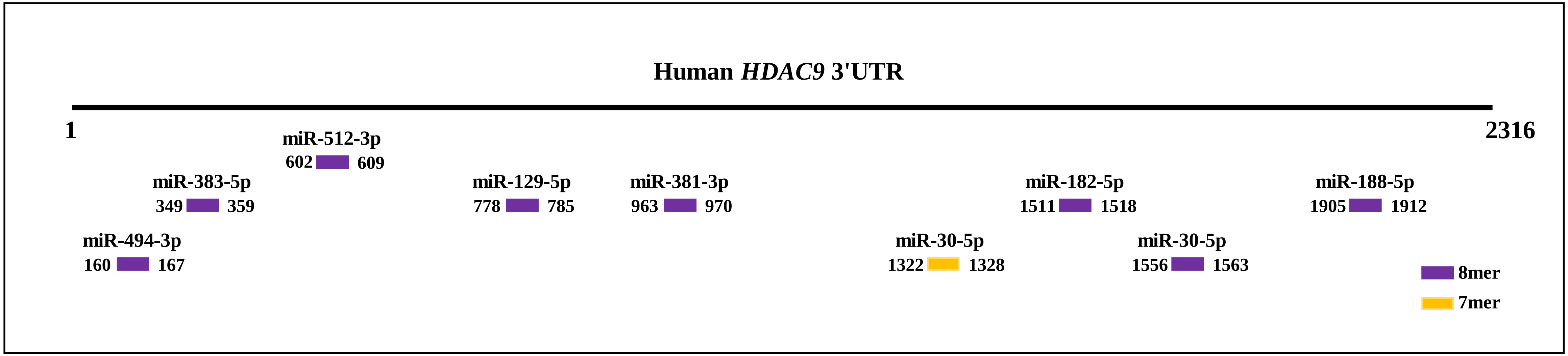
Human HDAC9 3'UTR
1
2316
miR-512-3p
602 609
miR-383-5p
349 359
miR-129-5p
778 785
miR-381-3p
963 970
miR-182-5p
1511 1518
miR-188-5p
1905 1912
miR-494-3p
160 167
miR-30-5p
1322 1328
miR-30-5p
1556 1563
8mer
7mer

Supplementary Table S1. The sequences of microRNA inhibitors and mimics

Name	Sequence (5'-3')
**Mimics**	
Negative control	*UUGUACUACACA AAAGUACUG*
miR-512	*CUGGAGUCGAUACUGUCGUGAA*

Supplementary Table S2. The sequences of SiRNAs

Name		Sequence (5'-3')
Negative control	sense	*UUCUCCGAACGUGUCACGUTT*
	antisense	*ACGUGACACGUUCGGAGAATT*
SiHDAC9	sense	*GAAAAUGAGACUUCGGUUU*
	antisense	*AAACCGAAGUCUCAUUUUC*
SiCAGE	sense	*CAGUGAACAGAGUGAUCAA*
	antisense	*UUGAUCACUCUGUUCACUG*
SiSOX2	sense	*GGACCGUUACAAACAAGGA*
	antisense	*UCCUUGUUUGUAACGGUCC*

Supplementary Table S3. Primer sequences for qRT-PCR

Name		Sequence (5'-3')
U6		*CGCAAGGATGACACGCAAATTC*
miR-512-3p		*CUGGAGUCGAUACUGUCGUGAA*
Actin	Forward	*TGGACTTCGAGCAAGAGATG*
	Reverse	*GAAGGAAGGCTGGAAGAGTG*
CAGE	Forward	*AAAGGAATGGGCCTGGGATG*
	Reverse	*GCCTCCCAGGAGTTGCAATA*
HDAC9	Forward	*AGTCTGCACCTTTGCCTCAG*
	Reverse	*AAACCGAAGUCUCAUUUUC*
